# Bandgap prediction of two-dimensional materials using machine learning

**DOI:** 10.1371/journal.pone.0255637

**Published:** 2021-08-13

**Authors:** Yu Zhang, Wenjing Xu, Guangjie Liu, Zhiyong Zhang, Jinlong Zhu, Meng Li

**Affiliations:** 1 Department of Computer Science and Technology, Changchun Normal University, Changchun, China; 2 College of Information Science and Engineering, Shenyang University of Technology, Shenyang, China; Universiti Teknologi Malaysia, MALAYSIA

## Abstract

The bandgap of two-dimensional (2D) materials plays an important role in their applications to various devices. For instance, the gapless nature of graphene limits the use of this material to semiconductor device applications, whereas the indirect bandgap of molybdenum disulfide is suitable for electrical and photo-device applications. Therefore, predicting the bandgap rapidly and accurately for a given 2D material structure has great scientific significance in the manufacturing of semiconductor devices. Compared to the extremely high computation cost of conventional first-principles calculations, machine learning (ML) based on statistics may be a promising alternative to predicting bandgaps. Although ML algorithms have been used to predict the properties of materials, they have rarely been used to predict the properties of 2D materials. In this study, we apply four ML algorithms to predict the bandgaps of 2D materials based on the computational 2D materials database (C2DB). Gradient boosted decision trees and random forests are more effective in predicting bandgaps of 2D materials with an R^2^ >90% and root-mean-square error (RMSE) of ~0.24 eV and 0.27 eV, respectively. By contrast, support vector regression and multi-layer perceptron show that R^2^ is >70% with RMSE of ~0.41 eV and 0.43 eV, respectively. Finally, when the bandgap calculated without spin-orbit coupling (SOC) is used as a feature, the RMSEs of the four ML models decrease greatly to 0.09 eV, 0.10 eV, 0.17 eV, and 0.12 eV, respectively. The R^2^ of all the models is >94%. These results show that the properties of 2D materials can be rapidly obtained by ML prediction with high precision.

## Introduction

The bandgap is a significant electronic property of certain materials that has been utilized for the fabrication of semiconductor devices. Moore’s law has dominated the chip manufacturing industry for over 50 years. However, Moore’s law is nearing its end, as reported by *Nature* [1]. One of the promising methods to prolong this era is to replace silicon, the most common material used to make chips, with other materials for next-generation electronics [2–4]. Owing to their unique nanosheet structure, large surface area, and extraordinary physical and chemical properties, atomically thin two-dimensional (2D) materials have attracted considerable attention since the successful isolation and characterization of graphene in 2004 [5]. Two-dimensional materials have become the most promising substitutes for silicon. It is to be noted that 2D materials with different structures have different electrical properties. For instance, intrinsic graphene is gapless, which makes it impossible to switch off [6–8]. This major shortcoming of graphene limits its range of potential device applications. Conversely, molybdenum disulfide (MoS_2_), which originates from the transition metal dichalcogenide (TMDC) family, is a semiconductor with an indirect bandgap of ~1.89 eV [9, 10]. In addition, hexagonal boron nitride (h-BN) is an insulator with a wide gap of ~ 5.9 eV [11, 12]. Therefore, considering the diverse properties of different 2D materials, the priority is to study the electrical properties of 2D materials used in manufacturing semiconductor devices.

Based on this motivation, it is anticipated that many theoretical and experimental studies have been conducted on their electronic properties [13–15]. Although the results of experimental studies are more reliable, it is difficult to carry out experiments on these thin materials owing to experimental constraints. Theoretical research is an effective alternative to experimental research. Over the past decades, conventional first-principles calculations have been a powerful tool for calculating the structures and properties of materials [16–18]. For example, they have been used to study the structural and electronic properties of perfect, doped, and defective 2D materials [19–21], the interaction between the substrate and 2D materials [22, 23], and the interaction between the contacts and 2D materials [24, 25]. Although the results calculated by the first-principles theory are usually consistent with experimental results, this method is computationally expensive and time consuming [26, 27].

Recent progress in machine learning (ML), a data-driven technique, has been effectively used for material research [28–37]. For instance, ML has been used in guiding chemical synthesis, assisting material characterization, and designing new materials [38]. In particular, ML methods are considered suitable for predicting a series of material properties. Cherukara et al. created the first atomic-level model using ML to accurately predict the thermal properties of stanene [39]. Dieb et al. employed ML to determine the most stable structures of boron-doped graphene [40]. Wan et al. developed a convolutional neural network (CNN) model to predict the thermal conductivity of porous graphene [41]. Dong et al. developed deep learning algorithms to predict the bandgaps of hybrids of graphene and h-BN with arbitrary supercell configurations [42]. Baboukani et al. presented an ML method for predicting nanoscale friction in 2D materials [43]. Moreover, ML interatomic potentials have shown outstanding efficiency in predicting novel materials [44, 45], lattice dynamics [46], estimating the thermal conductivity [47, 48], and exploring the phononic properties of 2D materials [49].

In this study, we employ random forest (RF), support vector regression (SVR), gradient boosted decision tree (GBDT), and multi-layer perceptron (MLP) models to predict the bandgaps of 2D materials based on the open C2DB database. First, eight elemental features were selected to train our ML models. The GBDT and RF models were found to be superior to the SVR and MLP with a prediction accuracy >90% and prediction RMSE of 0.24 eV, and 0.27 eV, respectively. In addition, when the bandgap without SOC is added into the elemental feature space, the prediction RMSE of GBDT, RF, SVR, and MLP decreases to 0.09 eV, 0.10 eV, 0.17 eV, and 0.12 eV, respectively. Furthermore, the prediction accuracy R^2^ of the four ML models is 98%, 98%, 95%, and 97%, respectively.

## Materials and methods

### Machine learning

Machine learning in this study was performed in Python 3.6 code with Scikit-learn frameworks for SVR, MLP, RF, and GBDT. All hyperparameters were optimized, and model performance was evaluated by grid search based on the averaged RMSE of the validation set.

In existing studies, most data used for the prediction of 2D materials were calculated based on the first-principles theory. Consequently, the accuracy of the prediction is easily challenged by the root cause. The data used here are from the C2DB, which is a common 2D material database [50]. The database contains approximately 4000 different 2D monolayer crystal structures. The dataset was split into training and test datasets. The training and validation datasets were 2817 and 313, respectively. For the SVR method, the prediction value for the bandgap is

$${\hat{f}}_{SVR}(x)=\sum_{i=1}^{N}(\hat{a}-\alpha^{*})K(x_{i},x)+b$$ (1)

where *K* is the kernel function used to measure the difference between the training data *x*_*i*_ and prediction data *x*.

The kernel function is the radial basis function (RBF) denoted by

$$K(x_{i},x)=\text{exp}(-\frac{{\left\|x_{i}-x\right\|}^{2}}{2\sigma^{2}})$$

with the width σ > 0.

The *b* can be obtained by solving the following Lagrange function

$$T(\omega ,b,\zeta_{i},\zeta_{i}^{*})=\frac{1}{2}{\left\|\omega \right\|}^{2}+C\sum_{i=1}^{N}(\zeta_{i}+\zeta_{i}{}^{*})$$ (2)

$$\text{s.t.}\;y_{i}-{\hat{f}}_{SVR}(x_{i})\leq \varepsilon +\zeta_{i}$$

$$\begin{array}{l} {\hat{f}}_{SVR}(x_{i})-y_{i}\leq \varepsilon +\zeta_{i}^{*}\\ \zeta_{i},\zeta_{i}^{*}\geq 0,i=1,\cdots ,N \end{array}$$

where *C* is the regularization parameter.

For the MLP model, the prediction value for the bandgap is

$${\hat{f}}_{MLP}(x)=\sum_{i=1}^{N}W_{o}^{ji}\phi (W_{l}^{ji}x_{i}+b_{l})+b_{o}$$ (3)

where *φ*(*z*) = *z* is the activation function, $W_{l}{}^{ji}$ and $W_{o}{}^{ji}$ represents the weight of the *j*th neuron in the *l*_th_ and output layers connected to the *i*th neuron in the *(l-1)*_th_ and *(o-1)*_th_ layers, respectively; *b*_*1*_,*b*_*0*_ are the biases of the hidden and output layers, respectively.

The weights *W =* (*W*_1_, *W*_2_,⋯ *W*_*m*_)^T^ are repeatedly updated to minimize the loss function

$$L(W_{1}\cdots W_{m})=\frac{1}{2}{\left\|{\hat{f}}_{MLP}(x_{i})-y_{i}\right\|}_{2}^{2}+\frac{\alpha }{2}{\left\|W\right\|}_{2}^{2}$$ (4)

where *a* > 0 is a non-negative tuning parameter that controls the magnitude of the penalty $\frac{\alpha }{2}{\left\|W\right\|}_{2}^{2}$.

The weight is updated by

$$W^{i+1}=W^{i}-\eta \frac{\partial L^{i}}{\partial W}$$ (5)

where *η* is the learning rate.

**Fig 1:**
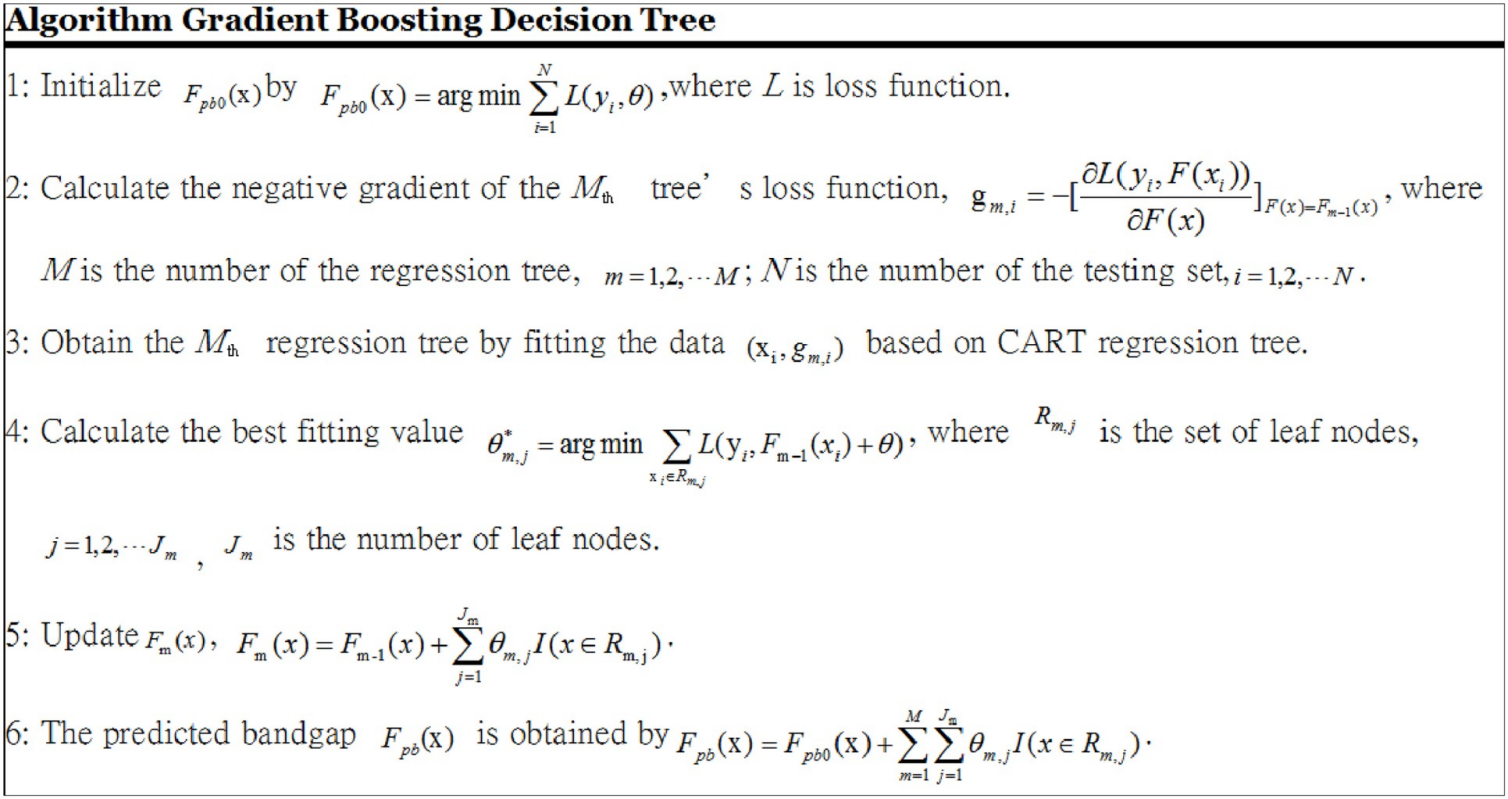
Gradient boosting decision tree algorithm.

**Fig 2:**
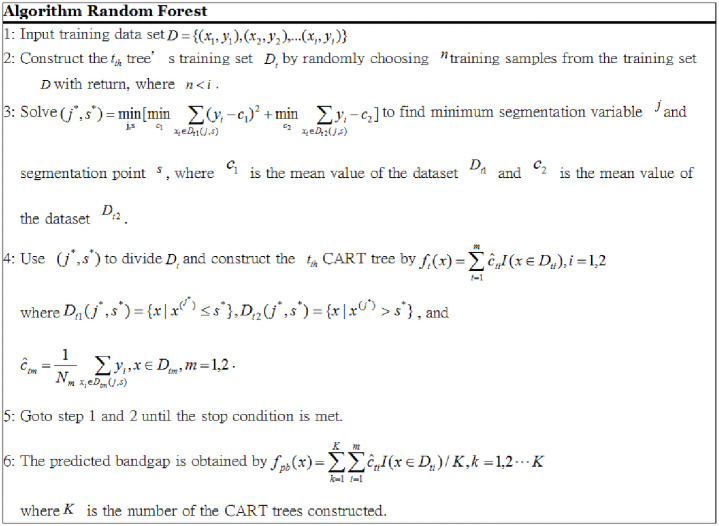
Random forest algorithm.

For the GBDT and RF models, the algorithmic details are provided in Figs 1 and 2.

### Criteria of evaluation

We used the mean absolute error (MAE), RMSE, and explained variance (R^2^) to evaluate the prediction accuracy of each model on the test set.


$$MAE=\frac{1}{k}\sum_{\text{i}=\text{1}}^{k}|y_{tb}^{i}-{\hat{y}}_{pb}^{i}|$$ (6)

$$RMSE=\sqrt{\frac{1}{k}\sum_{\text{i}=\text{1}}^{k}(y_{tb}^{i}-{\hat{y}}_{pb}^{i}{)}^{2}}$$ (7)

$$R^{2}=1-\frac{\sum_{\text{i}=\text{1}}^{k}(y_{tb}^{i}-{\hat{y}}_{pb}^{i}{)}^{2}}{\sum_{\text{i}=\text{1}}^{k}(y_{tb}^{i}-{\bar{y}}_{tb}^{i}{)}^{2}}$$ (8)

In formulas (6)–(8),${\text{y}}_{\text{tb}}^{\text{i}}$ is the true bandgap value randomly selected from the test set, ${\bar{y}}_{tb}^{i}$ is the average value of ${\text{y}}_{\text{tb}}^{\text{i}}$,${\hat{y}}_{\text{pb}}^{\text{i}}$ is the predicted value of the corresponding regression model, and *i* = 1,2,…,k, where k = 313.

### Spearman method

The spearman correlation coefficient is calculated by [51]

$$\rho =\frac{\sum_{i=1}^{N}\left(x_{i}-\bar{x})(y_{i}-\bar{y}\right)}{\sqrt{\sum_{i=1}^{N}{\left(x_{i}-\bar{x}\right)}^{2}}\sqrt{\sum_{i=1}^{N}{\left(y_{i}-\bar{y}\right)}^{2}}}$$ (9)

where X_*i*_ is the value of the *i*_th_ feature x in the training set, $\bar{x}$ is the average value of all the features, y_i_ is the value of the *i*_th_ feature y, $\bar{y}$ is its average value, and *i* = 1, 2, …, *N*, where *N* is the total number of samples in the training set.

## Results and discussions

Feature selection is a key step in ML algorithms. A good sample dataset can improve the performance of ML algorithms. The most common elemental information, including Dosef, Hform, Natoms, Mass, Cellarea, Energy, Smax, Fmax, and Volume, was first chosen for feature correlation analysis. The elemental features selected are listed in Table 1. The Pearson linear correlation coefficient map can analyze and identify features with high correlation, and eliminate the multiple collinearities between features, which leads to the distortion or inaccuracy of model estimation; this is particularly evident in linear models, such as SVR. Therefore, among many features with a strong correlation (correlation value greater than 0.8), only one effective feature is reserved. However, the Pearson correlation coefficient is not stable and is affected by the outlier values. Thus, scatter plots are also used. Scatter plots of features not only show outlier values but also show a high feature correlation. The scatter plots of the feature correlation and Pearson linear correlation coefficient map are provided in S1 and S2 Figs.

**Table 1:** Definitions of the features selected [50].

Feature Name	Definitions
Dosef	density of states at the Fermi energy
Hform	heat of formation
Natoms	number of atoms
Mass	sum of atomic masses in unit cell
Energy	total energy
Fmax	maximum force
Smax	maximum stress on unit cell
Volume	volume of unit cell
Gap_nosoc	gap w/o soc

**Fig 3:**
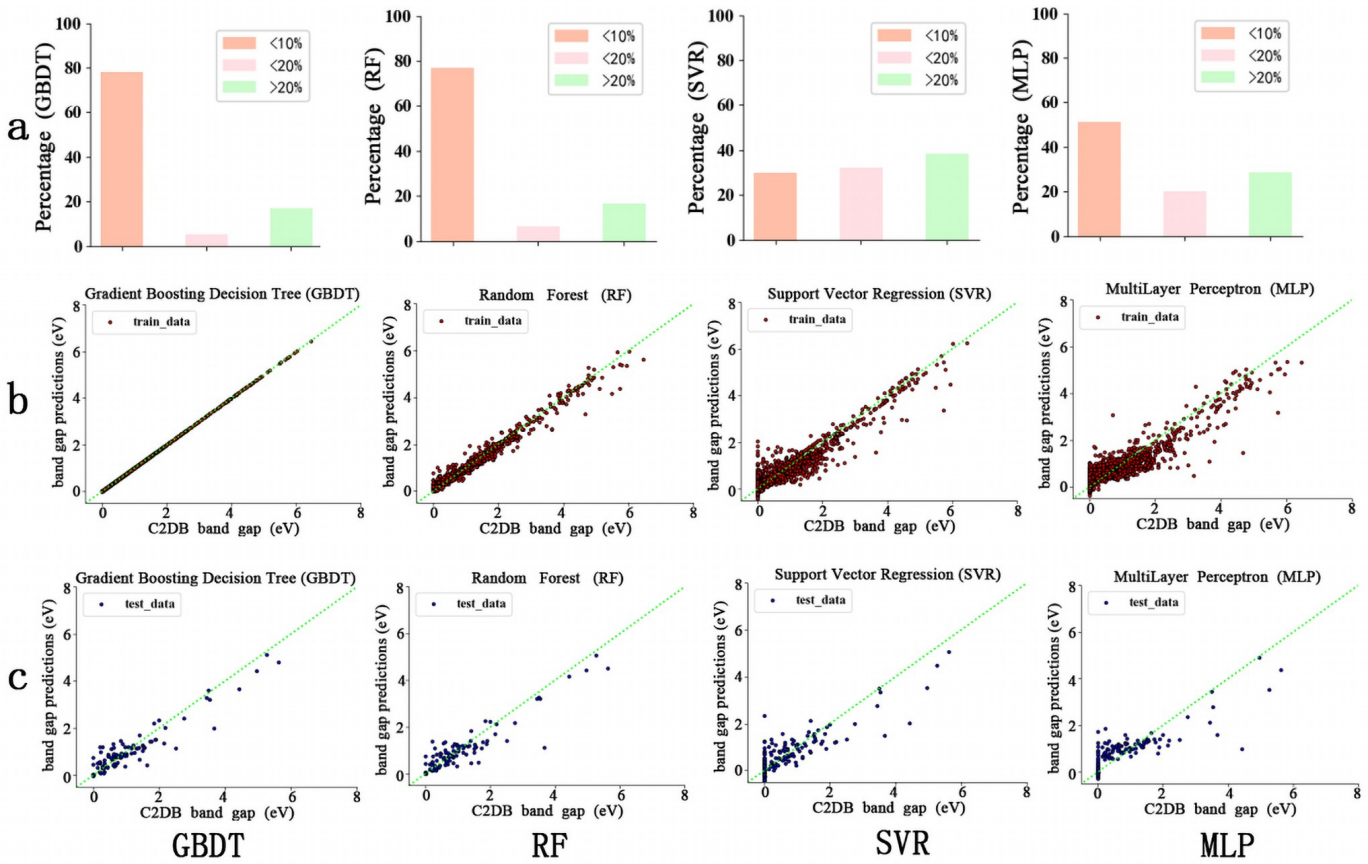
Prediction performance of four ML algorithms (GBDT, RF, SVR and MLP) without the feature Gap_nosoc. (a) Error levels of ML predicted bandgaps (b) Fitness between predicted bandgaps and true bandgaps on the training sets (c) Fitness between predicted bandgaps and true bandgaps on the test sets.

Four types of ML models: SVR, MLP, RF, and GBDT are used. The performance comparison of the four ML models with the 8-dimensional feature space (without Gap_nosoc) is shown in Fig 3. The prediction accuracy is characterized by the absolute error of the predicted bandgaps (*E*_ML_) to the C2DB bandgaps (*E*_C2DB_), which is calculated as |*E*_ML_−*E*_C2DB_|. As shown in Fig 3(a), RF and GBDT can predict the bandgaps within 10% absolute error for approximately 80% of the cases, whereas MLP shows approximately 50% of the cases. By contrast, the prediction results from SVR deviate much more from the C2DB benchmarks, showing >20% error for approximately 40% of the cases. Fig 3(b) and 3(c) show the fitness between the predicted bandgaps and true bandgaps on the training and test sets, respectively. The RF and GBDT exhibit a strong direct linear correlation between the predicted ML values and the C2DB values, whereas the SVM and MLP show weaker correlations. The predicted bandgaps of the four models for new materials with bandgap values between 4 and 6 showed relatively deviations, especially for SVR and MLP. This is because the amount of data with bandgap values between 4 and 6 in the training set is small. Consequently), the model cannot obtain a better prediction ability for new materials with bandgap values between 4 and 6 in the learning process.

**Fig 4:**
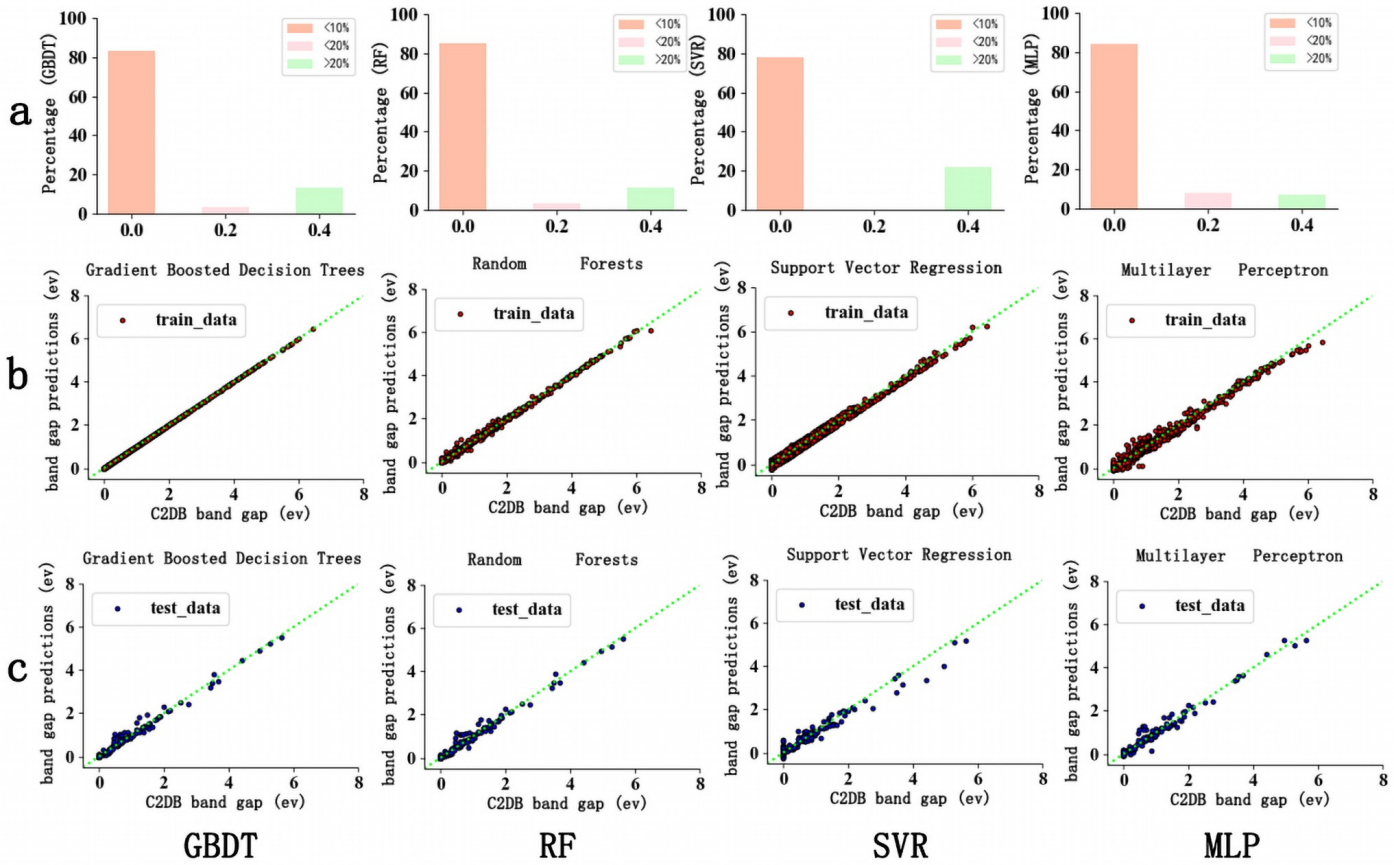
Prediction performance of four ML algorithms (GBDT, RF, SVR and MLP) with the feature Gap_nosoc. (a) Error levels of ML predicted bandgaps (b) Fitness between predicted bandgaps and true bandgaps on the training sets (c) Fitness between predicted bandgaps and true bandgaps on the test sets.

When the bandgap calculated without SOC was used as a feature, the performance of all four ML models was greatly enhanced, as shown in Fig 4. Fig 4(a) shows that the GBDT, RF, and MLP can predict the bandgap within 10% absolute error for >80% of the cases, and SVR shows >75% of the cases. The four ML models exhibit a very strong direct linear correlation between the predicted ML values and the C2DB values for both the training and test sets, as shown in Fig 4(b) and 4(c). The predicted bandgap values between 4 and 6 improved significantly. Only SVR showed relatively slight deviations.

Other indicators of the MAE, RMSE, and R^2^ were used to evaluate the regression performance of the ML models. Normally, the MAE and RMSE are much smaller and closer to 0, and R^2^ is greater and closer to 1, indicating better performance and higher prediction accuracy of the model. The MAE, RMSE, and R^2^ of the four ML models based on an 8-dimensional feature space are provided in Table 2. The GBDT shows the lowest RMSE of ~0.24 eV for the predicted bandgaps. The RMSE values slightly increase to 0.27 eV for the RF. By contrast, the prediction accuracy of the SVR and MLP algorithms is lower, showing higher RMSEs of 0.41 eV and 0.47 eV, respectively. R^2^ is an indicator of the correlation between the prediction and real values and is considered one of the most important metrics for evaluating the accuracy of the prediction models. Table 2 illustrates the bandgaps predicted by the GBDT and RF with ~92% and 90% relevance to the values from the C2DB database. Both SVR and MLP had >70% relevance. Both RF and GBDT are known to be more effective in predicting the bandgaps of 2D materials than other models. Among them, GBDT yields the best prediction results. This is because of the fusion of the decision tree and the gradient descent algorithm. Thus, the model has the advantages of high prediction accuracy, ability to deal with nonlinear data, flexibility to deal with various data types, stronger fitting ability, and better generalization ability for unknown new datasets. Table 3 shows the MAE, RMSE, and R^2^ of the four ML models based on a 9-dimensional feature space. The prediction precision was greatly improved when the bandgap calculated without SOC was added to the feature space. The bandgaps predicted by the four models had >94% relevance to the values from the C2DB database. The RMSE values of all the models fall drastically to 0.17 eV, 0.09 eV, 0.10 eV, and 0.12 eV, respectively. In summary, these results show that the RF and GBDT are superior to the SVR and MLP without highly relevant features in predicting the bandgaps of 2D materials. Provided that highly relevant features are chosen, ML models are less important and show high prediction accuracy.

**Table 2:** Statistics of predicted bandgaps by SVR, GBDT, RF and MLP algorithms based on an 8-dimensional feature space.

Models	Parameters	MAE	RMSE	R^2^
SVR	C = 50, epsilon = 0.2 gamma = 50,kernel = rbf	0.10	0.41	0.75
GBDT	n_estimators = 21000, max_depth = 21, min_samples_split = 5, max_features = 0.8, learning_rate = 0.001	0.12	0.24	0.92
RF	n_estimators = 15000, max_depth = 20, min_samples_split = 5,max_features = 0.8, min_samples_leaf = 3	0.10	0.27	0.90
MLP	solver = adam, hidden_layer_sizes = (262,140,139,180), activation = tanh,alpha = 1e-8, tol = 1e-6, max_iter = 5000, learning_rate_init = 0.01	0.24	0.43	0.73

**Table 3:** Statistics of predicted bandgaps by SVR, GBDT, RF and MLP algorithms based on a 9-dimensional feature space.

Models	Parameters	MAE	RMSE	R^2^
SVR	C = 50, epsilon = 0.2 gamma = 50,kernel = rbf	0.11	0.17	0.95
GBDT	n_estimators = 21000, max_depth = 21, min_samples_split = 5, max_features = 0.8, learning_rate = 0.001	0.03	0.09	0.98
RF	n_estimators = 15000, max_depth = 20, min_samples_split = 5,max_features = 0.8, min_samples_leaf = 3	0.03	0.10	0.98
MLP	solver = adam, hidden_layer_sizes = (262,140,139,180), activation = tanh,alpha = 1e-8, tol = 1e-6, max_iter = 5000, learning_rate_init = 0.01	0.06	0.12	0.97

**Fig 5:**
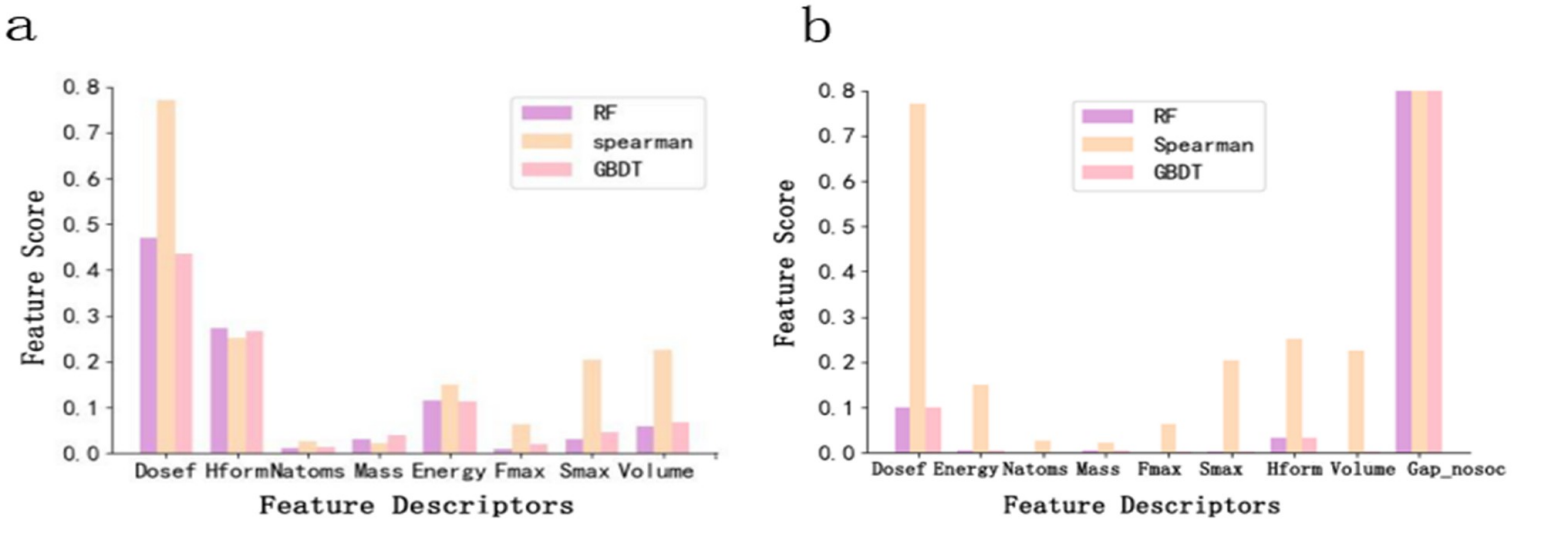
Feature importance evaluation. (a) 8-dimensional feature space (b) 9-dimensional feature space.

The correlation index between the features used for model training and the target was calculated by the Spearman method, which was used to judge the importance of features to the target. In addition, the RF and GBDT models have their feature importance scoring functions. Feature importance is shown in Fig 5. For the 8-dimensional feature space, as shown in Fig 5(a), the Spearman correlation coefficient indicates that the features of “Dosef,” “Hform,” “Volume,” “Smax,” and “Energy” have relatively high scores. For the RF and GBDT, the features of “Dosef,” “Hform,” and “Energy” have relatively high scores. For the 9-dimensional feature space, as shown in Fig 5(b), both “Dosef” and “Gap_nosoc” have the highest scores based on the Spearman correlation coefficient. For the RF and GBDT, the features of “Dosef,” “Hform,” and “Gap_nosoc” have relatively high scores. These results reveal that the features of “Dosef,” “Hform,” and “Gap_nosoc” have a high influence on the performance of the training model.

## Conclusion

Recently, researchers have performed ML to investigate the properties of 2D materials. Common databases of 2D materials are few and less developed. Therefore, most published studies used their calculated data to train ML models. The prediction accuracy of these data can be challenged by the root. Although all the data used here are available from the C2DB database, feature selection is more important. It is easy to train the models to obtain high prediction accuracy in other fields owing to the availability of a large amount of data; however, in the field of materials, data are very few and limited. Determining the highly relevant features from the limited datasets and training the models to obtain a high prediction accuracy is the key issue. In this study, four ML models trained on a common 2D material database were utilized to successfully predict the bandgap of 2D crystals. When Gap_nosoc is not added to the feature space, the GBDT and RF models yield prediction accuracy >90%, whereas the SVR and MLP models show lower prediction accuracy (70%). The RMSE of the GBDT is ~0.24 eV, whereas that of the SVR and MLP is ~0.41 eV. After Gap_nosoc is added to the feature space, the prediction accuracy of the four ML models is significantly improved. The RMSE of the four ML models decreases significantly to 0.09 eV, 0.10 eV, 0.17 eV, and 0.12 eV, respectively. The R^2^ of all the models is >94%. The results indicate that features highly relevant to the target have a major influence on the performance of the training model. In contrast to three-dimensional materials, the electrons in 2D materials are inherently constrained in the direction perpendicular to the material, which make 2D materials naturally have many unique properties. The bandgap is an essential electronic property of the materials. The width of the bandgap determines whether the material is a semiconductor or an insulator. Therefore, we first focused on predicting the bandgaps of 2D materials. In the future, we will investigate the prediction of other properties of 2D materials, such as magnetic properties, electron mobility, and mechanical stability.

## Supporting information

S1 Fig Scatter plot of the feature correlation. (DOCX)

S2 Fig Pearson linear correlation coefficient map. (DOCX)

S1 Data Availability of data and material. (RAR)

S1 Code Code used to build the model. (DOC)
