# Supplementary figures and images for: Bandgap prediction of two-dimensional materials using machine learning
Source: PLoS One. 2021 Aug 13;16(8):e0255637. doi: 10.1371/journal.pone.0255637 (PMC8363013; doi:10.1371/journal.pone.0255637)

**S1 Fig. Scatter plot of the feature correlation.**

**
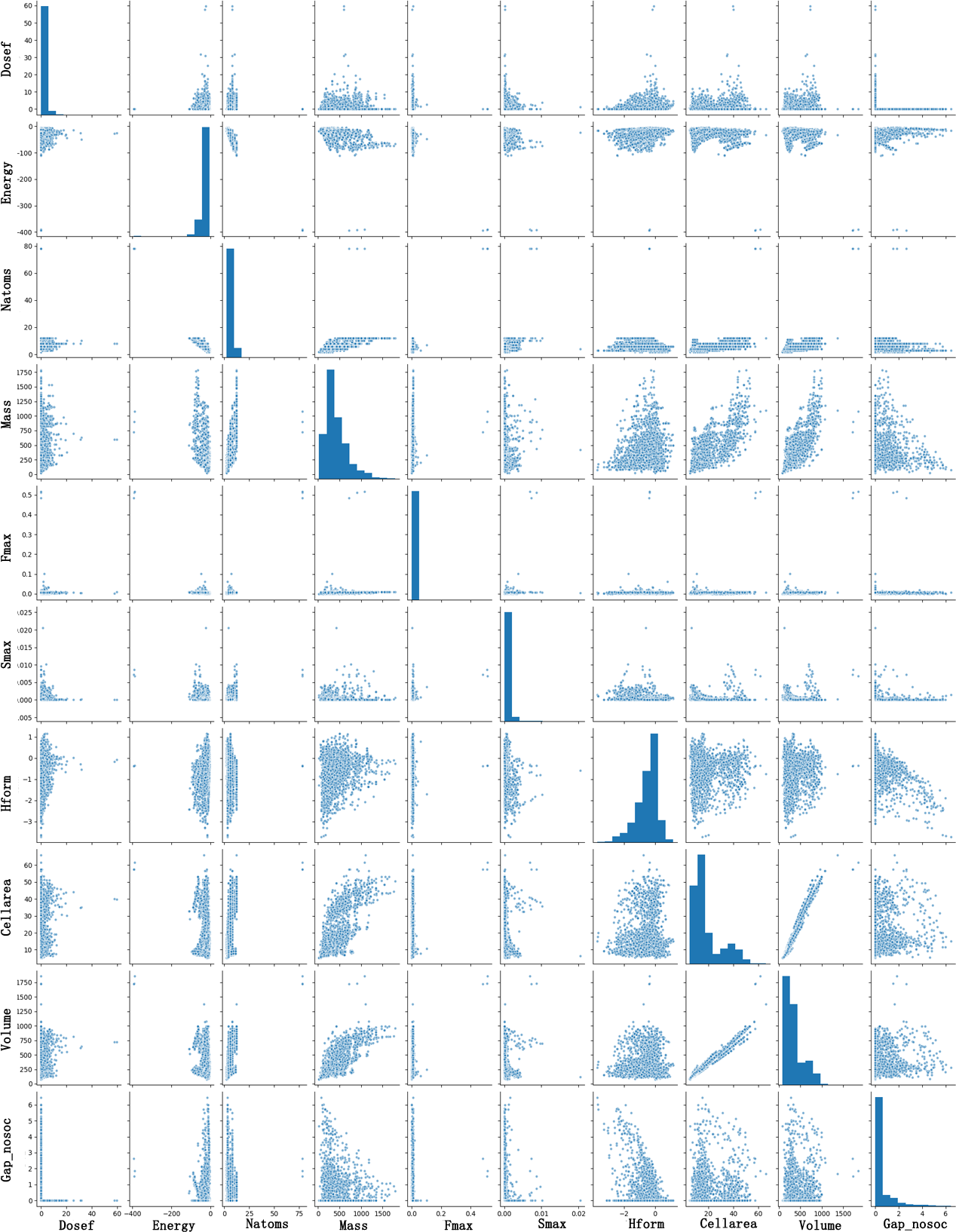
**

Supplement: S1 Fig — (DOCX) [file pone.0255637.s001.docx]
