# Supplementary material for: Bandgap prediction of two-dimensional materials using machine learning
Source: PLoS One. 2021 Aug 13;16(8):e0255637. doi: 10.1371/journal.pone.0255637 (PMC8363013; doi:10.1371/journal.pone.0255637)

**S2 Fig. Pearson linear correlation coefficient map.** As shown in S1 Fig, there are 45 subgraphs, representing the relationship between two features respectively. Therefore, only the scatter graph between the two features is a straight line and the two in the pearson linear correlation coefficient map(as shown in S2 Fig) are strongly correlated, a feature can be eliminated. As shown in S1 and S2 Figs, it can be observed that only the feature “cellarea” and volume meet the above conditions. Because the feature volume contains more information and is more conducive to model training, the feature “cellarea” is eliminated.


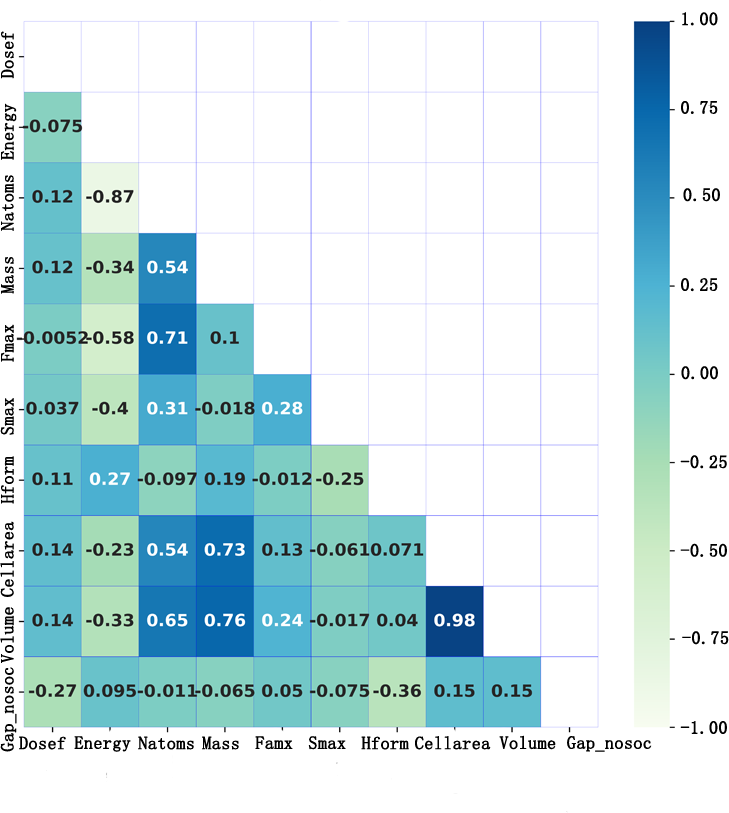

Supplement: S2 Fig — (DOCX) [file pone.0255637.s002.docx]
