# Supplementary material for: Bandgap prediction of two-dimensional materials using machine learning
Source: PLoS One. 2021 Aug 13;16(8):e0255637. doi: 10.1371/journal.pone.0255637 (PMC8363013; doi:10.1371/journal.pone.0255637)
Supplement: S1 Code — (DOC) [file pone.0255637.s004.doc]

Machine learning work in this paper was performed in Python 3.6 code with frame works Scikit-learn for support vector regression (SVR), multi-layer perceptron (MLP), random forests (RF) and gradient boosted decision trees (GBDT).

*# coding=UTF-8*

*#FileName: The model of SVR on R^2^, MAE and RMSE.py***from** sklearn.ensemble **import** RandomForestRegressor
**import** matplotlib.pyplot **as** plt
**from** sklearn.model_selection **import** GridSearchCV
**from** sklearn.svm **import** SVR
**from** sklearn **import** ensemble
**import** numpy **as** np
**import** pandas **as** pd
**from** sklearn.model_selection **import** train_test_split

**from** sklearn.metrics **import** mean_squared_error **from** sklearn.metrics **import** mean_absolute_error **from** sklearn.metrics **import** r2_score

*#  The absolute path to the csv file*data=pd.read_csv(**'C:/Users/ROCKXU/Desktop/dosfinal.csv'**)
df=data.values
df=np.array(df) **for** i **in** range(9):
 df[:,i]=(df[:,i]-df[:,i].min())/(df[:,i].max()-df[:,i].min())
data_train, data_test = train_test_split(df, test_size=0.1, random_state=0)
x_train=data_train[:,:9]
x_test=data_test[:,:9]
y_train=data_train[:,9]
y_test=data_test[:,9] kernel = **'rbf'**C = [50]
gamma = [50]
epsilon = [0.2]

*# Parameter dictionary*params_dict = {
 **'C'**: C,
 **'gamma'**: gamma,
 **'epsilon'**: epsilon
}
*# Grid parameter search*svr = SVR()
gsCV = GridSearchCV(
 estimator=svr,
 param_grid=params_dict,
 n_jobs=2,
 scoring=**'r2'**,
 cv=6
)
gsCV.fit(x_train, y_train)
svr = SVR(C=gsCV.best_params_[**'C'**], kernel=kernel, gamma=gsCV.best_params_[**'gamma'**],
 epsilon=gsCV.best_params_[**'epsilon'**])
svr.fit(x_train, y_train)
svr_train_pred = svr.predict(x_train)
svr_test_pred = svr.predict(x_test) print(**"Model evaluation - Test Set:"**)
print(**'r^2:'**,r2_score(y_test, svr_test_pred))
print(**'RSE'**, mean_squared_error(y_test, svr_test_pred))
print(**'RAE'**, mean_absolute_error(y_test, svr_test_pred))
print(**'RMSE:'**,np.sqrt(mean_squared_error(y_test,svr_test_pred)))

*# coding=UTF-8*

*#FileName: The model of RF on R^2^, MAE and RMSE.py***from** sklearn.ensemble **import** RandomForestRegressor
**import** matplotlib.pyplot **as** plt
**from** sklearn.model_selection **import** GridSearchCV
**from** sklearn.svm **import** SVR
**from** sklearn **import** ensemble
**import** numpy **as** np
**import** pandas **as** pd
**from** sklearn.model_selection **import** train_test_split

**from** sklearn.metrics **import** mean_squared_error **from** sklearn.metrics **import** mean_absolute_error **from** sklearn.metrics **import** r2_score data=pd.read_csv(**'C:/Users/ROCKXU/Desktop/dosfinal.csv'**)
df=data.values
df=np.array(df) **for** i **in** range(9):
 df[:,i]=(df[:,i]-df[:,i].min())/(df[:,i].max()-df[:,i].min())
data_train, data_test = train_test_split(df, test_size=0.1, random_state=0)
x_train=data_train[:,:9]
x_test=data_test[:,:9]
y_train=data_train[:,9]
y_test=data_test[:,9] rf=RandomForestRegressor(criterion=**'mse'**,bootstrap=**False**,max_features=0.8,
max_depth=20,min_samples_split=5, n_estimators=15000,min_samples_leaf=3,random_state=0)
rf.fit(x_train, y_train)
rf_train_pred=rf.predict(x_train)
rf_test_pred=rf.predict(x_test)

print(**"Model evaluation - Test Set:"**)
print(**'r^2:'**,r2_score(y_test, rf_test_pred))
print(**'RSE'**, mean_squared_error(y_test, rf_test_pred))
print(**'RAE'**, mean_absolute_error(y_test, rf_test_pred))
print(**'RMSE:'**,np.sqrt(mean_squared_error(y_test,rf_test_pred)))

*# coding=UTF-8*

*#FileName: The model of GBDT on R^2^, MAE and RMSE.py***from** sklearn.ensemble **import** RandomForestRegressor
**import** matplotlib.pyplot **as** plt
**from** sklearn.model_selection **import** GridSearchCV
**from** sklearn.svm **import** SVR
**from** sklearn **import** ensemble
**import** numpy **as** np
**import** pandas **as** pd
**from** sklearn.model_selection **import** train_test_split

**from** sklearn.metrics **import** mean_squared_error **from** sklearn.metrics **import** mean_absolute_error **from** sklearn.metrics **import** r2_score data=pd.read_csv(**'C:/Users/ROCKXU/Desktop/dosfinal.csv'**)
df=data.values
df=np.array(df) **for** i **in** range(9):
 df[:,i]=(df[:,i]-df[:,i].min())/(df[:,i].max()-df[:,i].min())
data_train, data_test = train_test_split(df, test_size=0.1, random_state=0)
x_train=data_train[:,:9]
x_test=data_test[:,:9]
y_train=data_train[:,9]
y_test=data_test[:,9] params = {**'n_estimators'**:21000, **'max_depth'**: 21, **'min_samples_split'**: 5,**'max_features'**:0.8,**'learning_rate'**: 0.001, **'loss'**: **'ls'**,**'random_state'**:0,**'subsample'**: 0.85}
gbr = ensemble.GradientBoostingRegressor(**params)
gbr.fit(x_train, y_train)
gbr_train_pred = gbr.predict(x_train)
gbr_test_pred = gbr.predict(x_test)

print(**"Model evaluation - Test Set:"**)
print(**'r^2:'**,r2_score(y_test, gbr_test_pred))
print(**'RSE'**, mean_squared_error(y_test, gbr_test_pred))
print(**'RAE'**, mean_absolute_error(y_test, gbr_test_pred))
print(**'RMSE:'**,np.sqrt(mean_squared_error(y_test,gbr_test_pred)))

*# coding=UTF-8
#FileName: The model of MLP on R^2^, MAE and RMSE.py***from** sklearn.neural_network **import** MLPRegressor **from** sklearn.preprocessing **import** StandardScaler
**from** sklearn.model_selection **import** train_test_split
**import** numpy **as** np
**import** matplotlib.pyplot **as** plt
**import** pandas **as** pd
**from** sklearn.metrics **import** mean_squared_error **from** sklearn.metrics **import** mean_absolute_error **from** sklearn.metrics **import** r2_score data = pd.read_csv(**'C:/Users/ROCKXU/Desktop/dosfinal.csv'**) data.head()
target = data[**'gap'**].values
features = data.drop(columns=[**'gap'**])
X = features
y = target
num = len(target)
y = y.reshape(num, 1)
print(y.shape)
X_train, X_test, y_train, y_test = train_test_split(X, y, test_size=0.1, random_state=0)
*# The training set of data is standardized, which greatly improves the accuracy*ss = StandardScaler()
X_train_scaled = ss.fit_transform(X_train)
X_test_scaled = ss.fit_transform(X_test) mlp_hw = MLPRegressor(solver=**'adam'**,hidden_layer_sizes=(262,140,139,180),activation=**'tanh'**,alpha=1e-8,
 tol=1e-6,max_iter=5000,learning_rate=**'constant'**,
 learning_rate_init=0.01,random_state=120)
mlp_hw.fit(X_train_scaled, y_train)
pred1_train = mlp_hw.predict(X_train_scaled)
y_pred = mlp_hw.predict((X_test_scaled)) print(**"Model evaluation - Test Set:"**)
print(**'r^2:'**,r2_score(y_test, y_pred))
print(**'RSE'**, mean_squared_error(y_test, y_pred))
print(**'RAE'**, mean_absolute_error(y_test, y_pred))
print(**'RMSE:'**,np.sqrt(mean_squared_error(y_test,y_pred)))
